# Lives of British Physicians

**Published:** 1830-10-01

**Authors:** 


### XXVIII.

### Lives of British Physicians.—Harvey.

We had only room to glance at this publication in some late articles, and introduce an extract from the life of Dr. Gooch. We shall probably, however, find that several passages in these lives may be capable of furnishing wholesome food for reflexion from time to time.

Speaking of Harvey, the biographer informs us that, though choleric and passionate in youth, that great anatomist was goodnatured and cheerful in manhood and age. His antagonists were treated with modest and temperate language—a great contrast to their own—and he ever praised those from whom he differed in opinion.

"He was a great martyr to the gout, and his method of treating himself was as follows:—He would sit with his legs bare, even if it were frosty weather, on the leads of Cockaine House, where he lived for some time with his brother Eliab, or put them into a pail of water, till he was almost dead with cold, and then he would betake himself to his stove, and so it was done. He was troubled with insomnolency, and would then get up and walk about his chamber in his shirt, till he was pretty cool, or even till he began to shiver, when he would return to bed and fall into a sleep."

But the more interesting topic relates to the practice of so great a man. He died worth 20,000 pounds—a sum not very large to be left by a court physician, who must have been at least 50 years in practice. One of his intimate acquaintances declared that he would not give threepence for Harvey's prescriptions, they were so complicated and heterogeneous—an error not now run into, but rather the reverse. The following passage might be made to bear upon some living characters—and still more upon some who have lately gone to their long homes.

"It is probable that Harvey was too much occupied in the pursuit of knowledge, too intent upon making discoveries in the world of science, to have cultivated the habit of quickly discriminating ordinary diseases, or to have become very expert and ready in the employment of the resources and expedients of the practical art of medicine. That his business declined after the publication of his doctrine of the circulation of the blood, he himself complained of, and ascribed to the opposition and jealousy of his rivals; but it is more likely that the habits of abstract speculation in which he now began to indulge caused him to neglect the usual arts of gaining the confidence of the public, which if a physician once possess, he needs not the countenance, and may boldly set at defiance the envy, of his professional brethren. The example of Harvey may be regarded, therefore, as a splendid illustration of the truth of the opinion of a late celebrated physician, as declared in his posthumous work: 'That the most successful treatment of patients depends upon the exertion of sagacity or good common sense, guided by a *competent* professional knowledge.' If anatomy alone were sufficient to make a great physician, who ever could have been put in competition with Harvey?"

We are inclined to think that there is some foundation for the popular notion, that he who is an ardent cultivator of any collateral science, or even of any one branch of the *elements* of medical science itself, is seldom a good practical physician or surgeon. The *reasoning* by which this notion might be supported must be based on the melancholy fact, that the longest life of attentive observation, at the bedside of sickness and death, is insufficient for the acquisition of an accurate knowledge of the phenomena of diseases and the operation of their remedies. The seduction of the auxiliary sciences, then, might be urged as a bar, or at least an impediment, to the highest advances in practical knowledge of which an individual was capable. That there is some truth in this train of reasoning there can be no doubt; and many dead as well as living examples might be quoted in illustration. But we fear that such examples and such reasonings are too eagerly laid hold of by the indolence of mankind to cloak ignorance—or rather to palliate its existence. We do not like therefore, to hear the auxiliary sciences despised by practical medical men; while, at the same time, we would advise those who are candidates for public patronage, to beware of dedicating more than a rigid and wise proportion of their studies to the auxiliary sciences—or to any one branch of elementary medical knowledge. A *distinction* gained in this way may prove gratifying to the feelings, but injurious to the professional interests of the individual.

We are inclined to believe that it was the incessant devotion to physiological pursuits, rather than the "jealousy of his rivals," that caused the declension of Harvey's practice. We cannot, indeed, judge accurately of the operation of the same causes in the time of Harvey, as compared with our own days. But, in the present state of things, the jealousy of rivals, we apprehend, goes for little in obstructing real talent. Patronage and favouritism may and do push people into notice, who, if left to their own resources would never be heard of; but we question whether the most rancorous hatred or jealousy can materially obstruct the progress of genenuine talent and undeviating *rectitude of conduct*. This last is *generally* indispensible to final and complete success. For although the CHARLATAN may fill his pockets, and the manœuvring REGULAR may gain a march on his more honest and honorable competitor, TIME usually dispels the illusion, and proves that "HONESTY IS THE BEST POLICY."

---

